# Intracranial pressure monitoring in the torture chambre

**DOI:** 10.5935/0103-507X.20150050

**Published:** 2015

**Authors:** Alberto Biestro

**Affiliations:** 1 Hospital de Clínicas, Faculdad de Medicina, Universidad de la República - Montevideo, Uruguay.

“What we learn to do, we learn by doing.”

Aristóteles (384-322 a.C.)

After the BEST TRIP study appeared in December 2012 in the New England Journal of Medicine,^(1)^ a large number of editorials, reviews, and new studies have addressed the issue of whether the monitoring of intracranial pressure (ICP) is relevant in the management of severe head injury and whether the costs are justified to achieve a better outcome. The article "Measurement of intracranial pressure and short-term outcome of patients with traumatic brain injury: a propensity-matched analysis" by Biselli-Ferreira et al.,^(2)^ published in this issue of RBTI, is yet another in a long series of accounts.

The study is a retrospective cohort study of patients with moderate and severe head trauma from a Brazilian hospital. The data were obtained from a computerized database; the sample consisted of 299 patients, of which only 28 were monitored for ICP (9.6%). Mortality was exceptionally low (16%). The patients with ICP and those without ICP were different in various aspects, and thus, the authors applied a technique of "matching" between the 2 populations and used the analytical method of estimated propensity,^(3)^ which is suitable when there are many variables to "match." Finally, 26 of the 28 patients with ICP and 26 patients without PIC who were well "matched" based on the predictors of the Crash megastudies were included in the study. The comparison of the outcomes of both samples comprising 26 patients each, form the basis of the study.

The authors found no differences between the two groups, with the exception of the length of stay of the survivors, which was approximately 6 days more in the patients with ICP (p < 0.05). Although the study seems made from a computer, running a database and away from bedside monitors and patients data sheet, we recognize that the conclusions the authors fit their findings.

Under these design conditions and with insufficient information available to the authors, we would not expect another results. Although it has been repeated ad nauseam, it is important remember that the measurement of ICP is a monitoring technique that, alone, cannot alter the outcome of any pathology. Any monitoring technique is inextricably linked with the concurrent therapies being implemented to determine the outcomes. Monitoring helps with rationalize the treatments and in applying them over a continual period of time.

The analysis technique of estimated propensity is a suitable statistical tool for observing the real world and approximating to the evidence, but it is entirely dependent on the variables we choose to "match." In this case, only prognostic factors established in other studies were used. Because the retrospective feature of study we completely do not know the details of therapeutic management that were effectively applied in both populations and as well as how they used the information provided by ICP monitoring. In a center with < 10% of patients with moderate and severe head injury monitored with ICP, there may have also been a significant shortfall in the management and interpretation of ICP measures, which were all as a result of the lack of trained personnel.

Apart from some criticism made^(4)^ at the time, we must agree that the BEST TRIP changed our perspective of ICP and its monitoring, and it currently continues being a topic of great interest. Twenty-three experts, including several BEST TRIP's authors, met in Seattle in 2013 and reached a consensus over 7 declarations about that study,^(5)^ 2 of which I wish to highlight: a) the knowledge of ICP should be deepen, and b) the practice of ICP monitoring should not be changed. Therefore, we are now studying various aspects of the ICP measure, such as the concept of intracranial pressure dose^(6)^ or the study of the treatment thresholds, which may vary from patient to patient and at different times throughout the course of the same patient. Another point to consider would be how identify by other variables the threshold of ICP to address all times.^(7)^

Finally, there is existing interest in developing treatments for intracranial hypertension, regardless of ICP monitoring, based on clinical and tomography images, which is also the underlying message of the study reviewed here. Although no one can deny the importance of clinical follow-up and imaging in the management of the trauma patient, these techniques are much closer to diagnostic procedures and are not strictly monitoring techniques (nor will ever be) because they cannot maintain a temporal continuum over the most important variable of intracranial hemodynamicin brain injury: specially at its initial stages when injury processes show considerable dynamics.
